# Surfactant Effects on Structure and Mechanical Properties of Ultrahigh-Molecular-Weight Polyethylene/Layered Silicate Composites

**DOI:** 10.3390/molecules22122149

**Published:** 2017-12-05

**Authors:** Leonid A. Nikiforov, Tatinana A. Okhlopkova, Iullia V. Kapitonova, Sardana A. Sleptsova, Aitalina A. Okhlopkova, Ee Le Shim, Jin-Ho Cho

**Affiliations:** 1 Department of Chemistry, North-Eastern Federal University, Yakutsk 677000, Russia; nikiforov_l@outlook.com (L.A.N.); botanya05@mail.ru (T.A.O.); Kirillinaiuliia@gmail.com (I.V.K.); ssard@yandex.ru (S.A.S.); 2 School of Mechanical & Automotive Engineering, Halla University, Wonju 220-712, Korea; elshim@halla.ac.kr; 3 Department of Chemistry, Myongji University, Yongin 449-728, Korea

**Keywords:** ultrahigh-molecular-weight polyethylene, alkyldimethylbenzylammonium chloride, low temperature, high strength

## Abstract

In this study, the reinforcement of ultrahigh-molecular-weight polyethylene (UHMWPE) with biotite was investigated. The biotite filler was mechanically activated with different dry surfactants to improve its compatibility with UHMWPE and decrease agglomeration among biotite particles. Alkyldimethylbenzylammonium chloride (ADBAC) and cetyltrimethylammonium bromide (CTAB) were selected as cationic surfactants. The tensile strength of composites containing 1 wt % of CTAB-treated biotite was increased by 30% relative to those with untreated biotite, but was unchanged with ADBAC treatment of the same biotite content. The stereochemistry of the surfactant may be critical to the composite structure and mechanical properties of the material. The stereochemistry of CTAB was preferable to that of ADBAC in enhancing mechanical properties because the stereochemistry of ADBAC impedes favorable interactions with the biotite surface during mechanical activation.

## 1. Introduction

The development of new functional polymer composite materials is of critical importance [1]. Anti-frost polymer composites are particularly important as the development of natural resources in arctic regions accelerates [2]. Although ultrahigh-molecular-weight polyethylene (UHMWPE) shows low friction coefficients, good mechanical properties, and high resistance to chemicals and frost, these properties are insufficient to meet the requirements for use in severe climatic conditions [3]. Many investigations have been performed to enhance the characteristics of UHMWPE required for use in extreme environments [4,5,6]. In particular, the application of layered silicates has attracted considerable interest [7,8,9,10], because they can be exfoliated in a polymer matrix due to the unique structure of the minerals. This interaction between the polymer and layered silicate provides a uniform distribution of filler in the polymer composite and maximizes the volume of the interfacial layer between the polymer and silicate. In addition, the polymer macromolecules can penetrate between the layers of the silicate to form an intercalated nanocomposite [11]. Both exfoliated and intercalated nanocomposites have enhanced mechanical and tribological properties, as well as unexpected characteristics such as decreased gas permeability, fire resistance, and electrical conductivity [12,13].

However, the polymer and layered silicate are not highly compatible. This incompatibility arises from the polarity differences between the materials. Layered silicate particles also easily agglomerate, which causes a decrease in surface energy and eventually degrades the mechanical properties of the composite [14,15].

The application of surfactants to the composite has been investigated to solve these problems [9,16]. Most composites composed of surfactant and layered silicates show high tensile strength, modulus, elasticity, and low friction coefficients [7,17,18,19,20,21]. In particular, Lee and colleagues improved the tensile strength and modulus by 62% compared to those of original polymer by using organoclay as a modifier of the polymer matrix [18]. However, previous investigations used solution-form surfactants to treat the layered silicates. No investigation of solid surfactant-treated layered silicates has been reported. In this study, the treatment of the silicate surface with a dry surfactant by joint mechanical activation in a planetary mill was performed, permitting the evaluation and discussion of surfactant effects on the mechanical properties of the composite.

## 2. Experimental Section

GUR-4120 UHMWPE (Ticona, Oberhausen, Germany) was selected as the polymer matrix because it has unique antifriction and wear-resistant properties [22,23]. As a filler, biotite or K(Mg,Fe)_3_[Si_3_AlO_10_(OH,F)_2_], from the Kuru-Vaara ceramic pegmatite mine in the Kovdor area of the Murmansk region in Russia, was used. Biotite occurs naturally and is less costly than synthetic nanofillers. The biotite was dried at 150 °C and then treated by joint mechanical activation in a planetary mill (Activator-2S, Activator, Russia) at 150 G [24,25] with cetyltrimethylammonium bromide ((C_16_H_33_)N(CH_3_)_3_Br, CTAB, Fluka Chemie Ag, Buchs, Switzerland) or alkyldimethylbenzylammonium chloride (ADBAC, Huntsman Corporation, The Woodlands, TX, USA) surfactants. In each mixture, 0.5 g of surfactant was added to 10 g of biotite.

**Figure 1:**
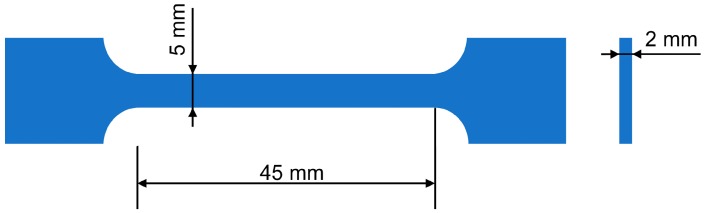
Schematic of the specimen used for the mechanical test.

The supramolecular structures of the composites were characterized by scanning electron microscopy (SEM, JSM-7800F, JEOL, Akishima, Japan). The tensile strength and relative elongation at break were measured according to the Russian state standard GOST 11262-80 (Autograph AGS-J, Shimadzu, Kyoto, Japan) at 25 °C. The strain rate and specimen length were 50 mm/min and 45 mm, respectively (Figure 1).

The degree of crystallinity, size of coherent scattering regions (CSR), and a number of micro-deformations in the crystalline structure of the polymer nanocomposites were estimated by wide-angle X-ray diffraction (XRD, ARL X’Tra, Thermo Fisher Scientific, Zug, Switzerland). The X-ray wavelength was fixed at 0.154056 nm. The scan angle ranged from 3° to 60° in steps of 0.04° and 0.02°, and the scanning rate was 1 s per point. WinXRD software was used for data analysis.

After mechanical activation in the planetary mill, the surfactant-modified biotite and UHMWPE were mixed in a paddle mixer. The resulting polymer composites were prepared by hot-pressing at 175 °C with a specific load of 10 MPa.

## 3. Result and Discussion

Mechanical activation increases the surface area as well as internal and surface energies, and decreases the coherence energy of solids [24]. The mechanical activation of the layered silicate with a surfactant causes the adsorption of the surfactant on the surface of the silicate. These physicochemical changes occur during the mechanical activation process. Recently, Khare and Burris reported that a certain amount of filler with a high surface energy caused the agglomeration of filler particles [14]. This agglomeration decreased the interfacial layer volume, which was critical to the reinforcement of the polymer composites. Eventually, it caused the degradation of mechanical properties, such as tensile strength and elongation at break. However, surfactants significantly inhibit the agglomeration of fillers [11].

**Figure 2:**
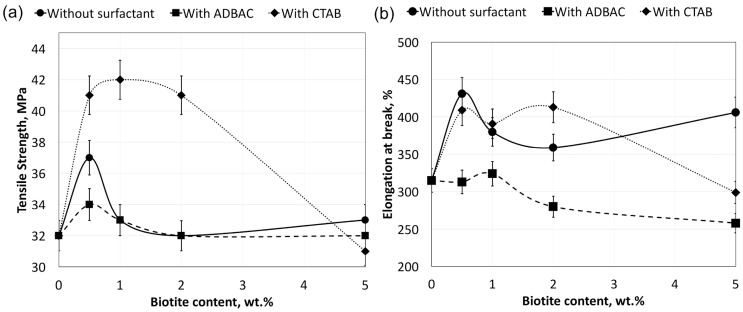
Mechanical properties of the polymer composites: tensile strength (**a**) and elongation at break (**b**). CTAB: cetyltrimethylammonium bromide; ADBAC: alkyldimethylbenzylammonium chloride.

The mechanical properties of the polymer silicate composites with and without surfactant, UHMWPE/biotite/(CTAB or ADBAC) and UHMWPE/biotite, are presented in Figure 2. The tensile strength of the UHMWPE/biotite/CTAB composite containing 1 wt % biotite is increased by 30% compared to that of the polymer matrix alone. With the exception of the 5 wt % biotite composite, all CTAB-modified composites show superior mechanical properties compared to those of the composites without surfactant. This is a result of increased adhesive interactions between the polymer matrix and CTAB-modified biotite [26]. As shown in Figure 2, the high tensile strength of the UHMWPE/biotite/CTAB composites with 0.5–2 wt % biotite can be explained by decreased agglomeration. The nature of the surfactant greatly affects the mechanical characteristics of the composites, as shown in Figure 2. ADBAC does not change the tensile strength and even decreases the elongation at break of the composites at all concentrations of biotite, compared to those with unmodified filler.

**Figure 3:**
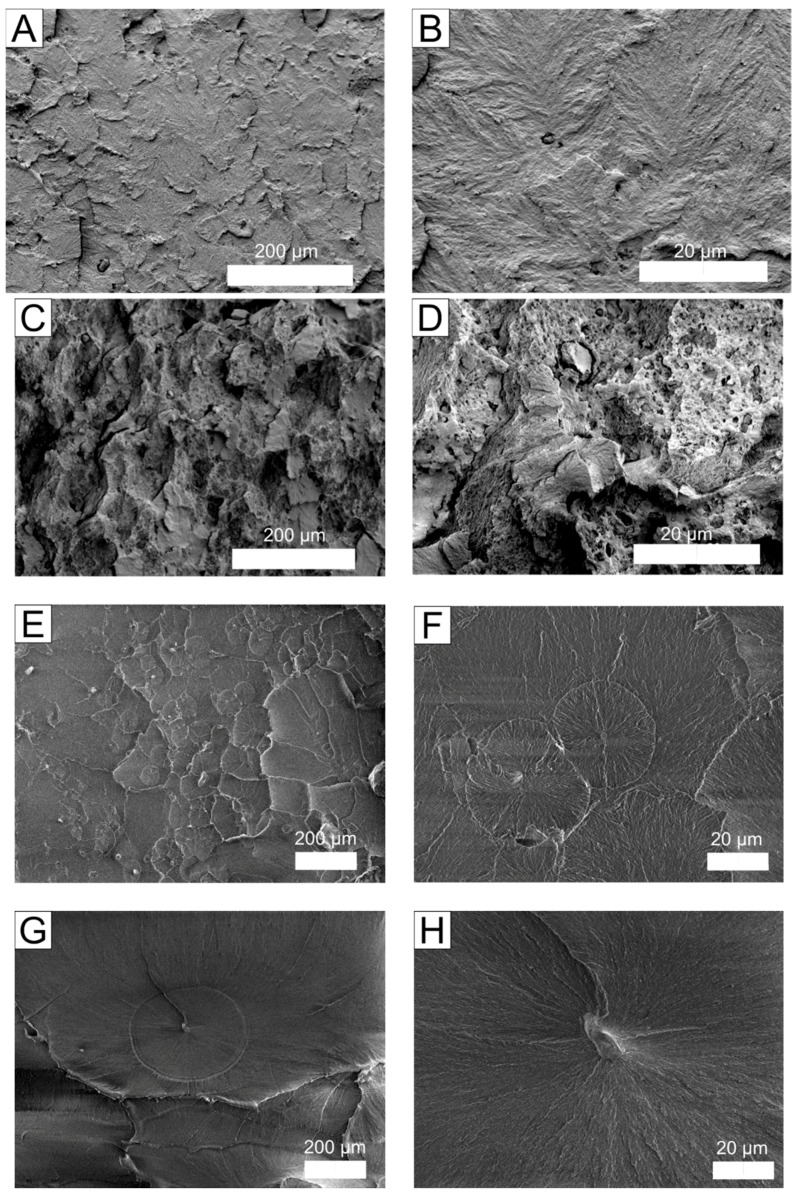
SEM micrographs of composites: (**A**,**B**) UHMWPE + 1 wt % biotite; (**C**,**D**) UHMWPE + 1 wt % biotite + CTAB; (**E**,**F**) UHMWPE + 1 wt % biotite + ADBAC; (**G**,**H**) unmodified UHMWPE. UHMWPE: ultra-high molecular weight polyethylene; CTAB: cetyltrimethylammonium bromide; ADBAC: alkyldimethylbenzylammonium chloride.

To further investigate the differences in mechanical properties, the microstructures of the composites were examined. SEM micrographs of the supramolecular structures of the composites with and without surfactants are presented in Figure 3. Figure 3 shows that the structures of the composites with surfactant differ from those without surfactant, indicating that the surfactant plays a significant role in composite formation. As shown in Figure 3c,d, many pores and irregular regions are formed in the composite with CTAB. The irregular structures of the CTAB-modified composites may arise from the formation of exfoliated or intercalated silicate layers, which are responsible for the enhanced mechanical properties [11]. All surfactants induce a reduction in size of the structural elements in the composites. The supramolecular structure of the ADBAC composites can be described as spherulitic. The morphology of the UHMWPE/biotite/ADBAC composite is similar to those of the UHMWPE/biotite composite and the initial polymer, indicating that biotite does not effectively interact with ADBAC. Therefore, ADBAC behaves not as a surfactant, but simply as a different species of filler.

The XRD patterns obtained from the polymer composites show that the degree of crystallinity is unaffected by the presence of CTAB for all filler concentrations, as shown in Table 1. This indicates that the biotite particles and CTAB do not participate in the crystallization process. During composite formation, the polymer molecules adsorbed on the biotite surface preferably form amorphous phases, rather than crystals. However, with ADBAC, the degree of crystallinity increases compared to the unmodified or CTAB-modified composites, but remains constant for all biotite concentrations. This indicates that ADBAC molecules may act as nucleation centers in the composite.

**Figure 4:**
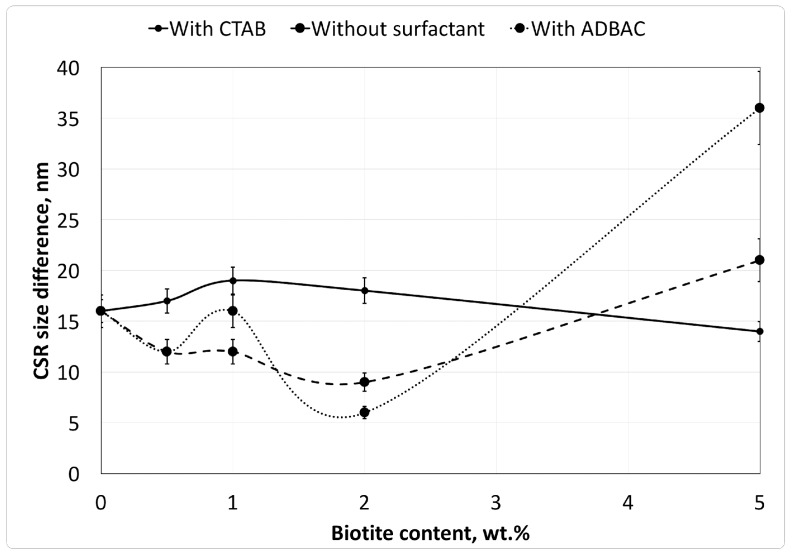
Changes in the coherent scattering region (CSR) size with different surfactant species as a function of biotite content in the polymer composites. CTAB: cetyltrimethylammonium bromide; ADBAC: alkyldimethylbenzylammonium chloride.

Microstructural parameters, such as the distortion level and the average CSR sizes, were evaluated from the XRD patterns using the Williamson–Hall method [27] and the Scherrer formula (Table 1). Peak broadening is due to both the micro-distortion of crystalline units and the average size of crystallites. The contribution of micro-distortion cannot be calculated by the Scherrer formula, but is provided by the Williamson–Hall method. Therefore, the difference between CSR sizes as calculated by the two methods corresponds to the contribution of micro-distortions to peak broadening in the XRD patterns. The relationship between CSR size differences and the biotite concentrations is shown for the different composites in Figure 4.

As shown in Figure 4, the crystalline structure of the composites exhibits higher micro-distortion, as indicated by the difference in the calculated CSR size, in the composites modified with CTAB than in those without surfactant.

**Figure 5:**
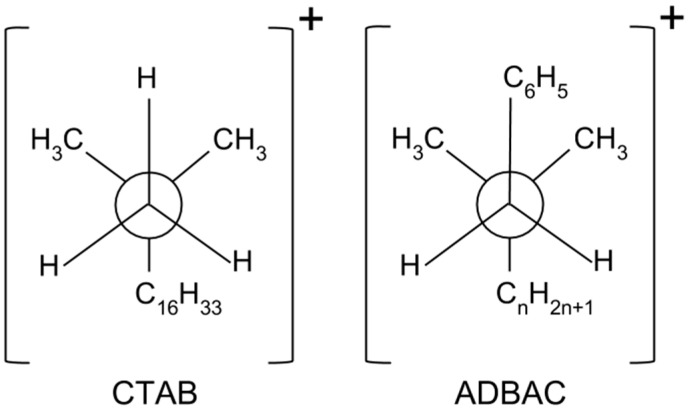
Newman projections of the surfactants (central circles indicate nitrogen atoms). CTAB: cetyltrimethylammonium bromide; ADBAC: alkyldimethylbenzylammonium chloride.

The different structures of the surfactant-modified polymer composites can be explained by the different stereochemistry of the surfactants. The Newman projections of the surfactants, which indicate the stable conformations of the carbon-nitrogen bonds in the surfactant molecules, are shown in Figure 5.

The positively charged nitrogen atom in ADBAC is blocked by the π electrons of the benzyl group, indicated by C_6_H_5_ in Figure 5, from the opposite long hydrocarbon chain (C_n_H_2n+1_). This stereochemistry impedes electrostatic interactions and cationic exchange between the hydrophilic center of the ADBAC molecule and the negatively charged silicate layers. However, the ammonium ion in CTAB is not blocked by large functional groups and interacts easily with the negatively charged silicate layers. It is likely that the ADBAC molecules are located in the UHMWPE matrix without contacting the silicate layers. Thus, they act not as surfactants, but as a filler species, which degrades the mechanical properties of the composites containing ADBAC.

## 4. Conclusions

Polymer composites were fabricated using biotite mechanically activated by solid surfactants to enhance the mechanical properties of the resulting materials. The chemical nature of the surfactant was crucial in improving the mechanical properties of the composites. In particular, steric factors in the solid-state surfactants defined the interactions between the silicate layers and the surfactant. The massive benzyl group in ADBAC interfered with the electrostatic interactions between the positively charged center and the negatively charged silicate surface.

## Figures and Tables

**Table 1:** X-ray diffraction data of polymer composites.

Sample	α, %	CSR Size (Williamson-Hall), nm	CSR Size (Scherrer), nm
Initial UHMWPE	54	30	14
UHMWPE + 0.5 wt % biotite	55	26	14
UHMWPE + 1 wt % biotite	53	27	15
UHMWPE + 2 wt % biotite	55	20	11
UHMWPE + 5 wt % biotite	50	37	16
UHMWPE + 0.5 wt % biotite + CTAB	53	32	15
UHMWPE + 1 wt % biotite + CTAB	52	33	14
UHMWPE + 2 wt % biotite + CTAB	54	34	16
UHMWPE + 5 wt % biotite + CTAB	53	31	17
UHMWPE + 0.5 wt % biotite + ADBAC	60	28	16
UHMWPE + 1 wt % biotite + ADBAC	60	31	15
UHMWPE + 2 wt % biotite + ADBAC	60	23	17
UHMWPE + 5 wt % biotite + ADBAC	62	52	16

Note: α—degree of crystallinity; UHMWPE: ultra-high molecular weight polyethylene; CSR: coherent scattering region; CTAB: cetyltrimethylammonium bromide; ADBAC: alkyldimethylbenzylammonium chloride.
